# Gender-based violence against female academics in Chile: organizational vulnerability, underreporting, and work-related impact

**DOI:** 10.3389/fpsyg.2026.1798923

**Published:** 2026-10-07

**Authors:** Vilbett Briones-Labarca, María Lourdes Campos, Angelo Araya-Piñones, Heidi Herrera-Ortega

**Affiliations:** 1 Department of Food Engineering, Universidad de La Serena, La Serena, Chile; 2 Department of Psychology, Universidad de La Serena, La Serena, Chile

**Keywords:** academic hierarchy, female academics, gender-based violence, institutional trust, job insecurity, organizational culture, organizational justice, underreporting

## Abstract

**Background:**

Few studies have examined, in an integrated way, the intersection of contract stability and rank and their relationship to (non) reporting and labor consequences, particularly in specific contexts, In Gender-based violence (GBV) in higher education. This study examined whether contractual conditions, academic rank, disciplinary unit affiliation, institutional trust, and cultural coping norms are associated with experiences of gender-based violence, reporting of such violence, and perceived impact on academic work among female academics in Chile.

**Methods:**

An analytic cross-sectional survey was conducted among female academics at a regional public university in Northern Chile. The final sample included 85 participants selected from a target population of 229 female academics. The questionnaire assessed self-reported GBV experiences during the last 5 years, including psychological, sexual, and physical violence; perpetrator groups; occurrence contexts; help-seeking and formal reporting; perceived effects on university life; knowledge of institutional regulations; and perceptions of organizational culture. Descriptive analyses, Fisher–Freeman–Halton exact tests, Kruskal-Wallis tests, and logistic regression models, including Firth penalized estimation, were used to examine organizationally patterned associations.

**Results:**

The 67% of participants (57/85) reported at least one GBV experience during the last 5 years, with psychological violence being the most frequent form, followed by sexual violence. Psychological violence varied significantly by faculty affiliation (*p* = 0.003), reaching 84% in Science and 83.3% in Engineering, and by contract type (*p* = 0.027), with the highest proportion among female academics with temporary contracts (80.5%). Academic staff were the most frequently identified perpetrators, accounting for 64.6 and 73.0% of the psychological and sexual violence mentions, respectively. Only 7% of those who reported experiencing situations of violence formally reported the incidents to the relevant units within the university, whereas 93% did not; the main reasons for not reporting by those who suffered some types of violence were the minimization of the incident (49%) and distrust of bureaucratic processes (22%). In the final logistic regression model, higher odds of reporting GBV were observed in Science (OR = 7.96, *p* = 0.003), Humanities (OR = 3.77, *p* = 0.043), and Engineering (OR = 9.88, *p* = 0.002) compared with Social, Business, and Law Sciences. Temporary contract academics also showed higher odds than fee-based academics (OR = 3.25, *p* = 0.051).

**Conclusion:**

GBV against female academics is not only an interpersonal problem but also an organizational and occupational issue shaped by disciplinary contexts, employment insecurity, academic hierarchies, and weak institutional trust. The results highlight the need for universities to strengthen reporting mechanisms, guarantee protection regardless of contract status, provide psychosocial and legal support, and implement follow-up systems that ensure due process independent of the victim’s faculty or contract type. These measures are particularly relevant for the effective implementation of Chilean Law 21.369 and the advancement of organizational justice in higher education.

## 1 Introduction

Violence against women is a global problem, with approximately 30% of women being victims of physical or sexual violence during their lives (UN Women, 2024). Gender-based violence constitutes a violation of Human Rights, threatening dignity, physical and moral integrity, equality, safety, and autonomy. This has negative consequences for people’s physical and psychological health, which is reflected in psychosomatic illnesses, anxiety, distress, exhaustion, sleep problems, eating disorders, a constant state of alertness, low self-esteem, stress, suicidal ideation, panic attacks and depression (Hourani et al., 2021; Porter and López-Angulo, 2022; Lausi et al., 2024; Vidal Palacios et al., 2024). In academic workplaces, these harms also translate into psychological and organizational outcomes, including impaired work functioning, reduced productivity, and withdrawal from professional opportunities. Framing gender-based violence as an organizational and occupational health issue is essential to understand underreporting and to inform prevention strategies grounded in institutional trust and organizational justice.

Universities are not immune to this form of violence, as they reflect the learning, values, attitudes and biases of the societies in which they are located (Hernández-Ramírez, 2021; de Souza et al., 2021). Likewise, they produce and reproduce power relationships that intensify gender inequities and privilege hierarchies, giving rise to women’s subordination (Varela Guinot, 2020). In this context, gender-based violence affects academic performance and the development of a professional career, which is reflected in low productivity and qualifications, abandonment of or expulsion from courses, degrees, jobs, insecurity in contractual and wage conditions, and loss of work/academic opportunities (Humbert and Strid, 2024a). However, far less is known about gender-based violence against female academic staff, particularly how contract type and academic rank operate as structural vulnerability factors that shape exposure, professional consequences, and the likelihood of formal reporting.

Gender-based violence is considered a critical social problem in the university context worldwide (Diéguez Méndez et al., 2020). Although a significant portion of the literature has historically focused on students’ perspectives—examining, for example, dating violence (Workye et al., 2023; Laz-Figueroa et al., 2025; Neves et al., 2024)—recent large-scale research has explicitly broadened this focus to include academic and administrative staff. A notable milestone is the European UniSAFE project, which generated robust evidence on the experiences of gender-based violence among university employees (Simonsson et al., 2024; Pilinkaite Sotirovic et al., 2024; Pilinkaite Sotirovic and Blazyte, 2024). In these diverse university populations, phenomena such as sexist attitudes (Rollero et al., 2019), the reproduction of stereotypes (Dinamarca Noack and Trujillo, 2021), microaggressions, and workplace violence have been observed (Gardiner and Finn, 2022; Tasqueto Tassinari et al., 2022; Cazares-Palacios et al., 2022; Humbert et al., 2025; Cavaleiro and Lizzi, 2024). However, while previous research has extensively examined gender-based violence in higher education, evidence specifically focused on female academics in Chile remains limited, particularly regarding the relationship between gender-based violence, academic hierarchy, working conditions, reporting, and perceived institutional responses. Regarding contract type and academic rank, there is little evidence of their impact on gender-based violence and its professional consequences in this regional context.

In Chile, the higher education system has historically been characterized by hierarchical and rigid structures that often normalize gender inequalities. The catalyst for dismantling these systemic inequalities was the Feminist May of 2018, a massive wave of student protests and university occupations across the country that denounced systemic sexual harassment and gender discrimination (Duarte and Rodríguez, 2022). This mobilization contributed to placing sexual harassment and gender-based violence in higher education on the national agenda and preceded the enactment of Law 21.369, which entered into force in 2021. The law mandates that Chilean higher-education institutions implement comprehensive policies, protocols, and prevention models to address gender-based violence (Chapa Romero et al., 2022; Suárez et al., 2024).

However, while this event successfully shifted the national agenda and forced the creation of formal reporting mechanisms, a gap remains between the existence of these regulatory frameworks and their effective capacity to protect academic staff within deeply ingrained hierarchical and precarious labor structures. This implementation gap is not only normative but also organizational: when reporting routes are perceived as opaque, costly, or unsafe, institutional trust erodes, and formal mechanisms remain underused. Therefore, examining how contractual insecurity and academic hierarchy shape perceived costs is essential. The incorporation of a gender perspective in university institutional work is based on international organizations’ guidelines, which reveal the role of gender equality as an instrument for development and gender transversalization as the most appropriate strategy to promote women’s rights (Suárez and Ganda Contreras, 2022). This approach is linked to Sustainable Development Goal (SDG) number 5 of the UN 2030 Agenda, which aims to “achieving gender equality and empowering all women and girls” (United Nations, 2018). This is supported by the Beijing Declaration and Platform for Action, originally adopted in 1995 (UN Women, 2015), and the Convention on the Elimination of All Forms of Discrimination against Women (CEDAW, 1979).

To fully comprehend the impact of these power asymmetries, it is essential to contextualize the current state of gender inequality in the Chilean university system. Despite recent institutional advances, academia in Chile remains characterized by pronounced vertical segregation and a persistent “glass ceiling” (Jorquera Torres, 2023). According to recent Gender Radiographies published by the Chilean Ministry of Science, Technology, Knowledge, and Innovation (MinCiencia, 2023, 2025), although women constitute approximately 44% of the overall academic staff, their representation drops drastically at higher hierarchical levels. The latest 2025 report highlights that while female participation in research has increased, significant gaps persist in STEM fields, and parity has yet to be achieved in formative academic spaces. Consequently, while there is relative parity at entry levels and in temporary or fee-based positions, women occupy less than 25% of Full Professor positions and remain significantly underrepresented in top-tier university leadership (SIES, 2023). This “leaky pipeline” reflects an organizational structure that not only concentrates formal power within male-dominated senior ranks but also perpetuates structural vulnerability for women academics in the early and most precarious stages of their professional careers.

In the context of demonstrations in Chile, female academics from the educational staff of the country have been of interest from a literature perspective. It has been reported that 87.5% of female academics have experienced sexual harassment or abuse (Carrasco, 2018); verbal harassment was the most frequent form (Universidad de Chile, 2019); high rates of sexual harassment and discrimination (Universidad Austral de Chile, 2018; SUR, 2022); and psychological (46%) and sexual (40%) violence (Universidad de Santiago de Chile, 2022) have been reported in the country. Beyond these cases, multiple Chilean university diagnostics have reported high rates of gender-based violence and harassment affecting female staff and students, with psychological and sexual forms being recurrently documented (Universidad de Los Lagos, 2019; Universidad de La Frontera, 2020; Universidad de Aysén, 2021; Pontificia Universidad Católica de Chile, 2023).

In the case of universities, this involves a set of actions and strategies aimed at incorporating global change into the institutional culture through the coordination of different strategic management mechanisms. This task involves two important challenges for the Commission on Gender Equality of the Council of Rectors of Chilean Universities (CRUCH, 2022): (1) facing a culture that is strongly rooted in sexist archetypes, both at an individual and collective level within universities, and (2) reviewing the policies contributing to the strengthening of practices that avoid violence, the reproduction of stereotypes, and gender discrimination to take measures to promote inclusion and diversity at an institutional level (Suárez et al., 2024). Therefore, we used the Kaleidoscope Model (CRUCH, 2022) as a conceptual framework to organize the domains assessed in this study and guide the interpretation of the findings.

In response to these challenges, various universities have implemented strategies to strengthen gender equality, reduce gender gaps, and prevent gender violence through policy creation. However, the future of these initiatives has not yet been mapped; therefore, there is no evidence supporting the approach and success of this system at the organizational and educational management level. In turn, local and international evidence has focused more on the student population, with a gap in information in the academic setting (for a review, see Nowrouzi-Kia et al., 2024), particularly among female academics.

Concerning the exposed background at the national and international levels, a relevant aspect of this study is the analysis of the different types of gendered violence perceived by female academics in the context of the functions they perform in the institution, which is essential for their professional development and improvement of their working conditions (Garcés et al., 2020). Understanding the specific challenges of gender-based violence faced by female academics contributes to strengthening practices within educational institutions to avoid producing and reproducing violence and power asymmetries (Buquet et al., 2020). They also contribute to improving selection, resource allocation, and promotion strategies in an inclusive and balanced environment (López et al., 2021).

A regional public university in northern Chile provides an analytically relevant setting for examining this problem. Following the social movement known as “Feminist May” in 2018, Chilean universities began to develop and strengthen institutional policies, protocols, prevention measures, and reporting mechanisms to address gender-based violence. These institutional developments were subsequently reinforced by Law 21.369, which came into force in 2021 and requires higher-education institutions to implement comprehensive measures to prevent, address, and sanction gender-based violence. However, these measures operate within academic environments characterized by contractual stratification and hierarchical career structures. Such conditions may increase employment dependency and the perceived professional costs of reporting gender-based violence, including concerns about possible retaliation and job loss.

By focusing on female academic staff in this Latin American context, the study contributes evidence on how employment precarity and rank operate as structural vulnerability factors shaping underreporting and work impact—an area that remains underrepresented in international literature.

Despite the growing body of literature on campus sexual conduct, research has disproportionately focused on student populations, leaving female academics’ experiences significantly under-theorized. Moreover, existing studies often treat gendered violence as an isolated interpersonal issue, overlooking how it is exacerbated by neoliberal restructuring of higher education. There is a critical knowledge gap regarding how contractual precariousness and flexible labor regimes, which are inherent to this model, create environments of vulnerability that facilitate violence and silence victims. This study moves beyond simple prevalence reporting to examine how these structural and contractual asymmetries function as institutional enablers of gendered violence within the academic hierarchy, aiming to bridge the gap in research on organizational determinants of victimization in higher education.

From an organizational psychology perspective, we conceptualize contract type and academic rank as structural indicators of dependency and exposure to retaliation costs, respectively. Therefore, we examined whether these organizational position variables are associated not only with victimization patterns but also with professional consequences, particularly perceived impacts on academic/scientific productivity and the (non)use of formal reporting channels. By linking gender-based violence to institutional trust and reporting behavior, this study extends the literature beyond prevalence estimates toward organizational-justice-relevant evidence that can inform structural interventions.

The central research question guiding this study is as follows: How are organizational conditions, including contract type, low academic rank, disciplinary unit of affiliation, institutional trust, and organizational culture associated with reported exposure to gender-based violence, formal reporting, and professional and personal consequences for female academics? We hypothesize that gender-based violence against female academics is associated with organizational vulnerability conditions, particularly contractual insecurity, academic rank, disciplinary affiliation, institutional trust, and shared cultural norms that minimize incidents or normalize discriminatory, verbal, symbolic, and sexualized behavior in academic contexts.

## 2 Methodology

### 2.1 Study design

This study used an analytic cross-sectional survey design (Frey, 2021) to examine gender-based violence experiences among female academics from four faculties of a public regional university in Northern Chile. The target population comprised all female academics employed by the institution at the time of data collection. The design was appropriate for identifying organizationally patterned associations between contract type, academic rank, faculty affiliation, and reported GBV experiences, but not for establishing causal relationships (Ahmed, 2024).

### 2.2 Participants and procedure

The study population consisted of all female academics employed at the institution at the time of data collection (*N* = 229). The final sample comprised 85 academics (*n* = 85), representing an overall response rate of 37.1% of the target population.

The inclusion criteria were: 1. Be over 18 years of age. 2. Teaching, research, and/or academic management functions are performed by the faculty. Belong to one of the university’s four faculties; 3. Have an active employment relationship formalized under one of the three contractual modalities analyzed: fees, temporary, or permanent; and 4. They must have a minimum of 3 years of seniority at their institution. Participation was strictly voluntary and anonymous.

A stratified probability sampling method with non-proportional allocation was used to select the sample. In this sampling design, the number of elements sampled from each stratum was not proportional to its representation in the total population. Elements of the population do not have the same probability of being included in the sample (Daniel, 2012). This strategy was deliberately chosen to ensure an adequate statistical representation of strata with lower population densities. Stratification was implemented based on two variables: faculty affiliation and contract type.

Regarding academic units, in the faculties with the largest number of staff (Humanities and Sciences), a minimum participation quota of 25% of their respective population was established; while in the faculties with the fewest female academics (Engineering and Social Sciences), a minimum threshold of 50% was set. Regarding contractual status, the following minimum participation percentages were determined by stratum: 25% for tenured staff, 40% for temporary staff, and 50% for permanent staff members. The decision to require greater coverage of temporary and permanent contracts stems from structural reasons within the university organization, given that these modalities guarantee regular physical, spatial, and time presence on campus.

Randomization was performed using Microsoft Excel. First, a complete list of eligible academics was compiled for each stratum, and a simple random selection algorithm (within each stratum) was applied to cover the planned allocation percentages of each stratum.

Of the target population (*N* = 229), 110 eligible female academics were initially contacted and invited. Of this group, 25 academics declined to participate or did not respond to the initial contact, resulting in a final sample of 85 participants. It is important to note that the original design did not include replacements for non-respondents to avoid altering allocation weights. The recruitment process is illustrated in Figure 1.

**Figure 1:**
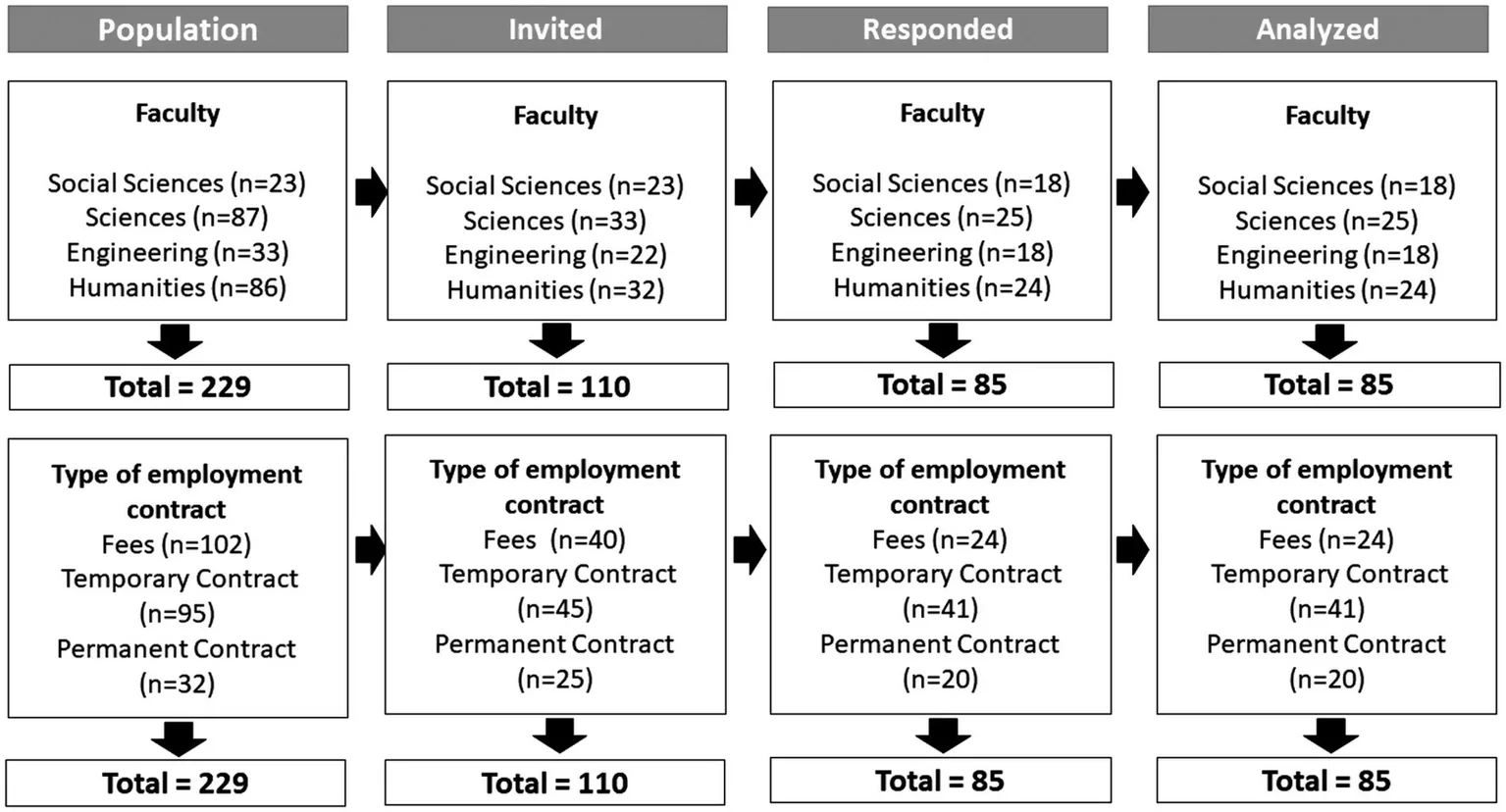
Participant flow diagram.

An analysis of non-response bias comparing the population with the final sample (Table 1) revealed significant differences in contract stability. While the most stable stratum (Permanent Contract) achieved a high proportional participation rate (20 of 32 academics; 62.5%), the most precarious stratum (fees) registered a significantly lower participation rate (24 of 102; 23.5%). This underrepresentation in the fee-based category does not invalidate the study; on the contrary, it is consistent with the literature on academic precarity, suggesting that job insecurity reduces the availability of time and willingness to participate in institutional surveys on sensitive issues such as sexual harassment. For the faculties, representation remained balanced according to the established minimum quotas.

**Table 1:** Sociodemographic and occupational characteristics of the sample.

Characteristic	Category	*n* (%)
Age group	Young	25 (29.4)
	Middle adult	24 (28.2)
	Older middle adult	36 (42.4)
Faculty	Sciences	25 (29.4)
	Social, Business and Law Sciences	18 (21.2)
	Humanities	24 (28.2)
	Engineering	18 (21.2)
Academic rank	Low	40 (47.1)
	Middle	35 (41.2)
	High	10 (11.8)
Contract type	Fee-based	24 (28.2)
	Temporary contract	41 (48.2)
	Permanent contract	20 (23.5)

The sociodemographic and occupational characteristics of the sample are shown in Table 1. Values are presented as frequencies and percentages of the final sample analyzed (*N* = 85).

Age group, academic rank, and contract type were grouped into analytical categories. Age group was categorized as young, middle adult, and older middle adult; academic rank as low, middle, and high; faculty affiliation as Social, Business and Law Sciences, Science, Humanities, and Engineering; and contract type as fee-based, temporary contract, and permanent contract. Gender identity is not displayed because all participants met the eligibility criterion of self-identifying as female academics.

Once the preliminary list was finalized, initial contact was made via institutional email. Academics who voluntarily agreed to participate signed an informed consent form. Subsequently, a face-to-face survey was administered, which lasted an average of 20 min.

The collected data were tabulated and organized in a digital format for subsequent descriptive analysis. Strict anonymity of the participants and confidentiality of the information were guaranteed. They were also informed of their right to omit questions or withdraw from the study at any stage without consequence.

### 2.3 Data collection form

Data were collected using the Diagnostic Survey on Gender-Based Violence, which was adapted from the Kaleidoscope Model developed by the Council of Rectors of Chilean Universities. The questionnaire comprised 19 main multipart items organized into five thematic sections (Universidad de Santiago de Chile, 2022): (1) sociodemographic and employment characteristics; (2) characterization of gender-based violence, perceived effects, and reporting; (3) knowledge of regulations and institutional response; (4) community effects; and (5) organizational culture and gender-based violence. The overall structure of the instrument is summarized in Figure 2, and the complete item organization and response formats are presented in Supplementary Table S1.

**Figure 2:**
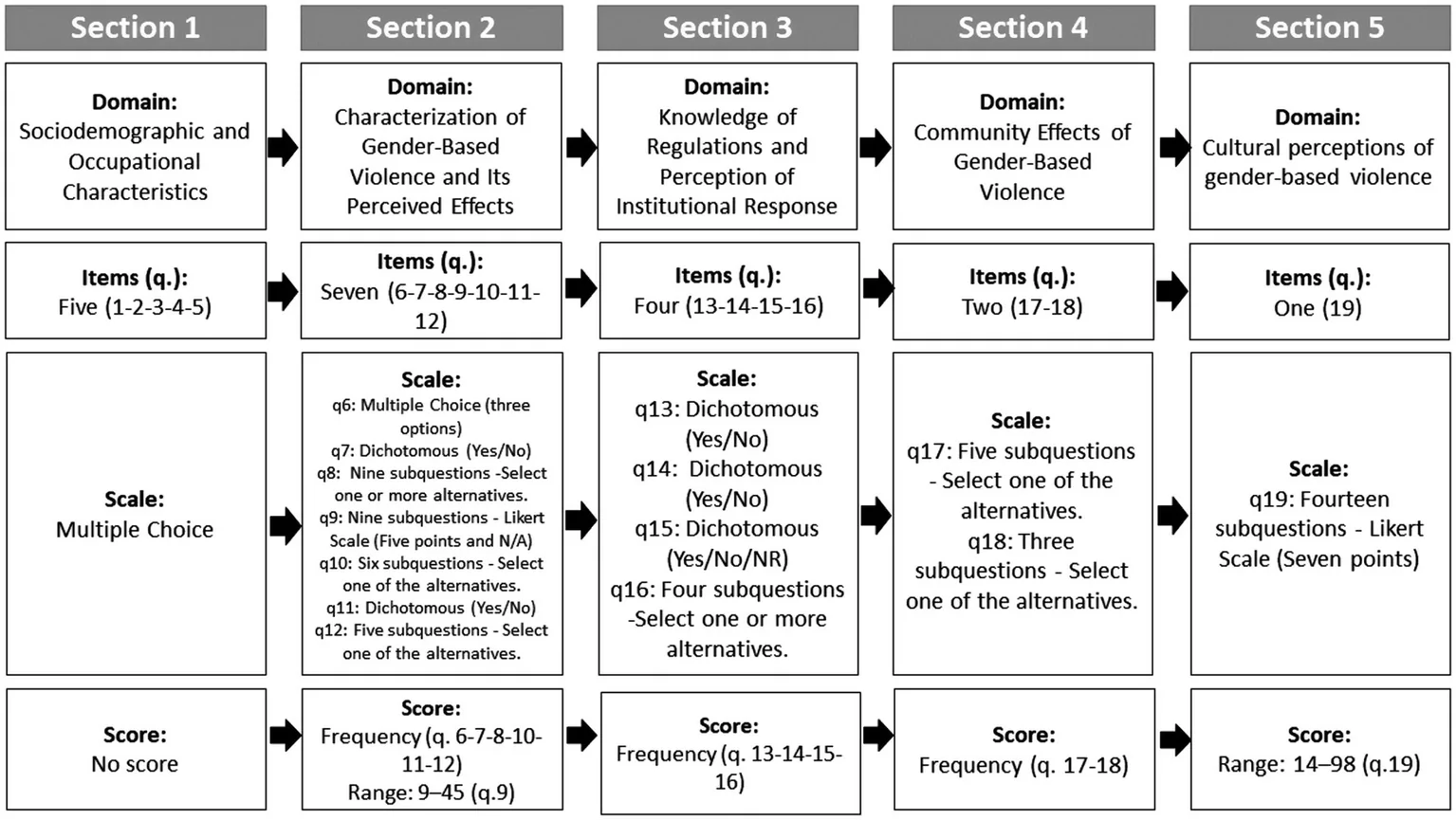
Survey structure and organization.

The questionnaire included binary, categorical, multiple-response, and Likert-type questions. Perceived effects were assessed using nine ordinal items rated on a five-point scale ranging from 1 (“slightly affected”) to 5 (“severely affected”), with an additional not applicable option. Organizational culture was assessed using 14 statements rated on a seven-point scale ranging from 1 (“strongly disagree”) to 7 (“strongly agree”). Items 6–12 included conditional questions and sub-items that were administered according to the participants’ previous responses. Multiple-response questions were treated as non-mutually exclusive categories. A comprehensive architectural mapping of these main items, their corresponding sub-items, response scales, and scoring ranges is visually summarized in Figure 2.

*Section 1*. Sociodemographic and employment characteristics (five items). Participants reported their age range, faculty, academic rank, and type of employment contract. These variables were used for analytical stratification to examine the differences associated with academic hierarchy and precarious employment status.

*Section 2*. Characterization of Gender-Based Violence and Its Perceived Effects (seven items): Exposure to gender-based violence was assessed by asking whether the participant had experienced any form of violence in the past 5 years. Those who answered affirmatively were asked to identify the perpetrator(s) (student, academic or administrative staff). The survey assessed specific behaviors, including discriminatory verbal/nonverbal comments, teasing, harassment, physical violence, leering, nonconsensual physical contact, sexual coercion, intimidation, and sexual assault. The contexts of occurrence (e.g., classrooms, offices, campuses, and social events) were recorded as multiple-choice items; therefore, these results are presented as frequencies of mention rather than mutually exclusive categories or estimates of individual prevalence. The perceived impact on professional life was assessed using nine items rated on a 5-point Likert scale (1: “slightly affected” to 5: “severely affected”), plus a “Not Applicable” option.

The items included physical and psychological distress, isolation from the team, absenteeism, decreased academic/scientific productivity, loss of opportunities and reassignment within the university. The total impact score was calculated by summing the nine items (range: 9–45), with higher scores indicating a greater perceived impact. The survey employed skip logic: participants who did not report experiencing violence were directed at the first question in Section 3.

*Section 3*. Knowledge of regulations and perceptions of institutional responses (four items). Knowledge of institutional regulations was assessed using dichotomous (yes/no) question. Participants also indicated which institutional responses they considered appropriate (e.g., legal support, mental/physical health support, awareness campaigns, and disciplinary sanctions).

*Section 4*. Community effects of gender-based violence (two items): These items assess cognitive and emotional reactions to gender violence (e.g., reliving past situations, anger/distrust, vulnerability, or perception of lack of impact).

*Section 5*. Cultural perceptions of gender-based violence (one item): Organizational culture regarding gender-based violence was measured using 14 statements rated on a 7-point Likert scale (1 = “strongly disagree” to 7: “strongly agree”). The items assessed perceptions of institutional safety, silence norms, causal attributions, trust in authorities and institutional justice. The total score was calculated by adding the 14 items (range: 14–98), with higher scores indicating a greater degree of agreement with the statements.

#### 2.3.1 Instrument validity

The survey underwent content validation through an expert review by five external experts (Elangovan and Sundaravel, 2021) to ensure conceptual adequacy, item clarity, and theoretical alignment with the CRUCH Kaleidoscope Model. The following criteria were established for selecting the evaluation panel: (1) holding a doctoral or master’s degree; (2) having academic or professional experience in the subject matter (gender-based violence); (3) having at least 5 years of experience in gender studies research in higher education; and (4) possessing knowledge of psychometrics or instrument design.

The validation procedure consisted of an independent evaluation round using a standardized rubric, in which each item and sub-item was assessed on a 1–4 scale. The review focused on the correspondence between each item and the intended construct (typologies of gender-based violence, perceived professional impact, and dimensions of organizational culture), as well as the cultural and linguistic appropriateness of the wording in the context of Chilean universities. Based on the feedback gathered, specific revisions were made to the original instruments. These included rewording three items in Section 2 to eliminate ambiguities, adding the “instructor” category to the hierarchy variable, ordering the faculties in the unit section, and ensuring the appropriateness of concepts (semantics) throughout the instrument. These modifications ensured that the instrument adequately covered the required domains and maximized its face and content validity prior to its application.

An exploratory reliability analysis was conducted on the organizational culture items used in this study. Based on their conceptual content, four items were grouped into an Institutional Support and Responsiveness scale: institutional awareness that gender-based violence is unacceptable, knowledge of where to seek help within the university, perceived institutional support for affected individuals, and perceived fairness of institutional measures and sanctions. Internal consistency was assessed using Cronbach’s alpha and McDonald’s omega. Reliability was considered adequate (Cronbach’s α = 0.747; McDonald’s ω = 0.768), and a composite score was computed as the mean of the four items, with higher values indicating more favorable perceptions of institutional support and responsiveness to sexual violence.

Owing to the cross-sectional design, sample size, and single-institution scope, this evidence should be interpreted as supporting content validity and internal consistency for this study rather than establishing full construct validity or generalizability across higher education settings.

#### 2.3.2 Interpretative considerations

To ensure an accurate interpretation of the findings, the questionnaire data were reported according to the measurement levels and response structures of each section. The individual prevalence of gender-based violence was estimated using items that asked whether participants had experienced any specific acts of violence during the last five years. This indicator was calculated based on a binary outcome variable, which took the value of one if participants reported at least one experience of gender-based violence and zero otherwise.

Items allowing multiple responses were analyzed as non-mutually exclusive responses. Accordingly, the results are presented as frequencies, percentages, or counts of mentions for each response option, and the total may exceed the number of respondents.

Likert-type items were treated as ordinal variables. These items were summarized using medians and interquartile ranges, and subgroup comparisons were conducted using non-parametric tests when appropriate. This approach was adopted to reduce the overinterpretation of sparse response categories and align the analysis with the distributional characteristics of the data.

Given the cross-sectional design, all analyses were interpreted as descriptive associations or group differences without causal inferences. “Not applicable” and missing responses were retained for descriptive reporting and were handled according to the requirements of each analysis using available-case procedures when applicable.

### 2.4 Data analysis

Analyses were performed using RStudio software. RStudio was used for data management, descriptive analyses, exact tests for categorical variables, nonparametric subgroup comparisons, reliability analyses and logistic regression models. Statistical significance was set at α = 0.05. Given the modest sample size and the presence of sparse cells in several contingency tables, the inferential results were interpreted cautiously and considered exploratory when necessary.

#### 2.4.1 Descriptive analysis

Categorical variables were summarized using frequencies and percentages. These included sociodemographic and employment characteristics, perpetrator group, specific manifestations of gender-based violence, occurrence contexts, help-seeking behavior, formal reporting, knowledge of institutional regulations, and perceived institutional responses. For multiple-response items, including specific behaviors, perpetrator groups, occurrence contexts, and institutional responses, results were reported as counts of mentions or as non-mutually exclusive percentages; consequently, totals may exceed the number of participants in the study. Likert-type outcomes were summarized using medians and interquartile ranges [IQR], consistent with the ordinal nature of the response scales and revised statistical tables. These outcomes included the perceived effects on university life and organizational culture.

#### 2.4.2 Categorical association analysis

Associations involving categorical variables were examined using the Fisher–Freeman–Halton exact test. These analyses were used to compare psychological, physical, and sexual violence according to the analysis categories established for age, faculty affiliation, academic rank, contract type, and the perpetrator group. Results for categorical comparisons are reported as frequencies, percentages, and exact test *p* values.

#### 2.4.3 Comparative analyses of Likert-type outcomes

Group differences in Likert-type outcomes were evaluated using the Kruskal-Wallis test. These analyses compared age groups, academic rank categories, and contract types for perceived effects on university life and selected organizational culture indicators. Results are reported as medians and interquartile ranges [IQR], together with Kruskal-Wallis *p*-values.

#### 2.4.4 Reliability analysis of institutional culture items

To examine perceptions of institutional culture regarding gender-based violence, an analysis of the organizational culture items was conducted separately. Differences across age groups, academic rank, and contract type were evaluated using the Kruskal-Wallis test because of the ordinal nature of the variables and the relatively small sample size. Medians and interquartile ranges (IQR) are reported. Additionally, an exploratory reliability analysis was conducted on the items related to organizational culture in the questionnaire. Based on their conceptual content, four items were grouped into an Institutional Support and Responsiveness scale, including: institutional awareness that gender-based violence is unacceptable, knowledge of where to seek help within the university, perceived institutional support for affected individuals, and perceived fairness of institutional measures and sanctions. Internal consistency was assessed using Cronbach’s alpha and McDonald’s omega.

#### 2.4.5 Multivariate analysis

A multivariate analysis was conducted to explore the factors associated with reporting at least one instance of gender-based violence. A binary outcome variable was constructed and coded as 1 when participants reported at least one experience of gender-based violence during the previous 5 years and 0 otherwise. The independent variables considered in the initial model were age group, academic rank, faculty affiliation, and contract type of faculty members. A full logistic regression model was estimated. To identify the most parsimonious specification, a bidirectional stepwise variable selection procedure based on the Akaike Information Criterion was applied. Faculty affiliation and contract type were retained in the final model. Odds ratios (ORs) and 95% confidence intervals (CIs) were estimated to quantify the strength and precision of these associations. Because the sample size was modest and some cells were sparse, a penalized logistic regression model using Firth’s bias-reduction method was also estimated to reduce the potential small-sample bias in the maximum likelihood estimates. The results of the standard and penalized logistic regression models were compared to assess their robustness. In accordance with the cross-sectional design, these models were interpreted as exploratory associations rather than causal effects.

#### 2.4.6 Missing data

Missing responses and “not applicable” categories were retained for the descriptive analysis. For inferential analyses, available case procedures were used, excluding missing values only from the specific analyses in which they occurred. No imputations were performed on the data.

### 2.5 Ethical aspects

The study was approved by the University Research Ethics Committee (Chile) (Approval No. 14/24) and conducted in accordance with the American Psychological Association (APA) ethical guidelines. All participants were contacted, their eligibility was confirmed, and written informed consent was obtained prior to data collection. Participation was voluntary, and the participants were informed that they could decline to answer any question or withdraw at any time without any consequences.

To protect confidentiality, the responses were recorded and analyzed in an anonymized format and were used exclusively for research purposes. Given the sensitive nature of gender-based violence, the survey was administered individually in a private setting to minimize discomfort, and the participants were informed about the available institutional support resources if the questionnaire elicited any distress.

## 3 Results

The Results are presented in four subsections: (i) characterization of gender-based violence in the university setting and its effects, (ii) knowledge of regulations and perceptions of institutional response, (iii) perceptions of organizational culture associated with gender-based violence, and (iv) multivariate analysis of factors associated with gender-based violence.

### 3.1 Characterization of gender violence in the university setting and its effects

Table 2 presents the distribution of psychological, physical, and sexual gender-based violence according to age group, teaching affiliation, academic rank, and contract type. The distribution of gender-based violence analyzed in this study establishes that psychological and sexual violence are the most prevalent forms of violence in the academia. Differences according to unit and contract type reveal that exposure is unevenly distributed among various organizational macro-units and affects female academics with more vulnerable contractual conditions than their male counterparts. Physical violence had the lowest incidence rate. These results indicate that gender-based violence in the academic field predominantly manifests as discriminatory, verbal, symbolic, and sexualized behaviors rather than physical aggression.

**Table 2:** Distribution of gender-based violence types by participant characteristics.

Characteristic	Category	*N*	Psychological violence, *n* (%)	*p*-value	Physical violence, *n* (%)	*p*-value	Sexual violence, *n* (%)	*p*-value
Age group	Young	25	18 (72.0)	0.147	4 (16.0)	0.217	13 (52.0)	0.222
	Middle adult	24	19 (79.2)		3 (12.5)		11 (45.8)	
	Older middle adult	36	20 (55.6)		1 (2.8)		11 (30.6)	
Faculty	Social, Business and Law Sciences	18	6 (33.3)	0.003	0 (0.0)	0.068	2 (11.1)	0.073
	Science	25	21 (84.0)		4 (16.0)		15 (60.0)	
	Humanities	24	15 (62.5)		1 (4.2)		8 (33.3)	
	Engineering	18	15 (83.3)		3 (16.7)		10 (55.6)	
Academic rank	Low	40	27 (67.5)	0.647	2 (5.0)	0.154	17 (42.5)	0.824
	Middle	35	22 (62.9)		6 (17.1)		15 (42.9)	
	High	10	8 (80.0)		0 (0.0)		3 (30.0)	
Contract type	Fee-based	24	12 (50.0)	0.027	0 (0.0)	0.141	7 (29.2)	0.305
	Temporary contract	41	33 (80.5)		6 (14.6)		20 (48.8)	
	Permanent contract	20	12 (60.0)		2 (10.0)		8 (40.0)	

Values are presented as numbers of participants and row percentages within each category. P-values correspond to Fisher–Freeman–Halton exact tests. Psychological violence included discriminatory comments, discriminatory jokes, other verbal or nonverbal discriminatory behaviors, and the publication of images or private conversations without consent. Sexual violence included leering or persistent sexualized looks, non-consensual approaches or physical contact, threats or pressure for sexual favors, sexual harassment, and sexual abuse or rape.

The age-based analysis revealed that psychological violence was reported by 72.0% of young, 79.2% of middle-aged, and 55.6% of older middle-aged academics. Sexual violence was also more frequently reported by young and middle-aged academics, at 52.0 and 45.8%, respectively, compared with 30.6% among older academics. Physical violence was less frequent in all age groups, ranging from 2.8% among older middle-aged academics to 16.0% among young academics. However, these age-related differences did not reach statistical significance for the perpetration of psychological violence.

Marked differences were observed in faculty affiliation. Psychological violence differed significantly across faculties (*p* = 0.003), with the highest proportions reported in Science and Engineering, where 84.0 and 83.3% of participants, respectively, reported experiencing it. Humanities showed an intermediate proportion of 62.5%, whereas Social, Business and Law Sciences showed the lowest proportion at 33.3%. Sexual violence also presented the highest proportion in Science (60%) and Engineering (55.6%), compared to 33.3% in Humanities and 11.1% in Social Sciences, Business and Law. Physical violence also showed higher proportions in the sciences and engineering than in the social sciences, business, and law, but these differences did not reach statistical significance.

Contract type was significantly associated with psychological violence (*p* = 0.027). Participants with temporary contracts reported the highest proportion of psychological violence (80.5%), compared to 60.0% among those with permanent contracts and 50.0% among fee-based academics. Sexual violence was also more frequent among participants with temporary and stable contracts; only academics with fee-based contracts showed a lower incidence, although these differences were not statistically significant. These results indicate that temporary employment is associated with a higher burden of psychological and sexual violence and may represent an organizational vulnerability in the academic workplace.

Table 3 describes the distribution of reported violence typologies according to the perpetrator group. As participants could report more than one perpetrator, the values represent the number of mentions rather than mutually exclusive individual cases. Across perpetrator groups, academic staff accounted for the largest proportion of perpetrators of psychological and sexual violence, at 64.6 and 73.0%, respectively. A significant association was observed between the perpetrator group and physical violence (*p* = 0.048), where the academic, administrative, and student staff accounted for 45.5, 36.4, and 18.2% of physical violence mentions, respectively. Academic staff were the most frequently identified perpetrator group, particularly for psychological and sexual violence, highlighting the relevance of peer-to-peer workplace relationships in the configuration of gender-based violence experience.

**Table 3:** Gender-based violence by perpetrator group.

Perpetrator group	Psychological violence, *n* (%)	*p*-value	Physical violence, *n* (%)	*p*-value	Sexual violence, *n* (%)	*p*-value
Student	39 (24.7)	0.296	2 (18.2)	0.048	12 (16.2)	0.325
Academic staff	102 (64.6)		5 (45.5)		54 (73.0)	
Administrative staff	17 (10.8)		4 (36.4)		8 (10.8)	

Values are counts of mentions, and column percentages. Participants could report multiple perpetrators and violence types; therefore, the totals refer to mentions, not mutually exclusive cases. P-values correspond to Fisher–Freeman–Halton exact tests.

Table 4 presents the specific manifestations of gender-based violence reported by participants. The most frequent behaviors were psychological ones. Discriminatory jokes were reported by 62.4% of participants, gender-based discriminatory comments by 58.8%, and other verbal or nonverbal discriminatory behaviors by 57.6% of participants. These findings confirm that psychological violence is largely expressed through everyday verbal, symbolic, and interactional practices that may normalize gender inequality within academic work environments. Sexualized behaviors were also frequently reported. Leering or persistent sexualized looks were reported by 51.8% of participants, making this the most common form of sexual violence. A non-consensual approach or physical contact was reported by 25.9% of participants. Less frequent but more severe forms of sexual violence were also present, including sexual harassment (reported by 4.7%), threats or pressure for sexual favors (reported by 3.5%), and sexual abuse or rape (reported by 1.2%). Physical violence was reported by 12.9% of participants. Publication of images or private conversations without consent, classified as psychological violence, was reported by 7.1% of participants.

**Table 4:** Specific manifestations of gender-based violence.

Type of violence	Specific behavior	*n* (%)
Psychological	Discriminatory jokes	53 (62.4)
Psychological	Gender-based discriminatory comments	50 (58.8)
Psychological	Other verbal/non-verbal discriminatory behaviors	49 (57.6)
Sexual	Leering or persistent sexualized looks	44 (51.8)
Sexual	Non-consensual approach or physical contact	22 (25.9)
Physical	Physical violence	11 (12.9)
Psychological	Publication of images or private conversations without consent	6 (7.1)
Sexual	Sexual harassment	4 (4.7)
Sexual	Threats or pressure for sexual favors	3 (3.5)
Sexual	Sexual abuse or rape	1 (1.2)

Values are n and percentages of the total sample [*N* = 85]. Multiple responses were allowed; therefore, the percentages are non-mutually exclusive and do not sum to 100%. The response categories were non-mutually exclusive because participants could select more than one behavior. Frequencies and percentages represent mentions of specific reported conduct and may exceed the number of participants. The listed behaviors are descriptive indicators and should not be interpreted as mutually exclusive. “Sexual harassment” was included as a separate row because it was a distinct response option in the questionnaire; it may conceptually overlap with other reported sexual behaviors such as leering, non-consensual physical contact, threats for sexual favors, and sexual abuse or rape.

Because participants could select more than one response, these percentages represent reported mentions; therefore, the findings should be interpreted as a descriptive profile of the reported manifestations of gender-based violence rather than as independent prevalence estimates for distinct categories.

The pattern observed in Table 4 indicates that gender-based violence among female academics was not limited to isolated severe events but included a continuum of discriminatory, symbolic, sexual, and coercive behavior. The predominance of discriminatory jokes, comments, and nonverbal discriminatory behaviors suggests the presence of normalized practices that may contribute to a hostile academic environment. Simultaneously, the presence of non-consensual contact, sexual harassment, pressure for sexual favors, and sexual abuse or rape indicates that more severe forms of gender-based violence were also reported, albeit at lower frequencies. As respondents could report more than one experience, the percentages were non-mutually exclusive and should not be summed to 100%.

Tables 2–4 show that psychological violence was the most common typology, followed by sexual violence. Significant differences were observed in terms of faculty affiliation and contract type related to psychological violence, and academic staff were the most frequently identified perpetrator group for both psychological and sexual violence. These results support an organizational interpretation of gender-based violence in academia, in which exposure is related not only to individual characteristics but also to disciplinary contexts, working conditions and peer relationships.

#### 3.1.1 University contexts in which gender-based violence occurred

Among the participants who reported experiencing gender-based violence (*n* = 57), 127 mentions of contexts in which gender-based violence occurred were recorded in the data. Because the questionnaire allowed for the mention of more than one context at a time, the results are presented as percentages of mentions per context. Therefore, the totals may exceed the number of respondents and should not be interpreted as mutually exclusive individual cases.

The reported experiences were concentrated in routine academic and work-related settings. The most frequently mentioned contexts were in-person academic contexts (71.9%), university offices (63.2%), university campus spaces (28.1%), and virtual academic contexts (19.3%).

By age group, context mentions were concentrated among middle-aged and older middle-aged academics, particularly in in-person academic contexts and university offices where teaching, supervision, research, and institutional interactions occur.

#### 3.1.2 Effects of gender -based violence on university life

Table 5 and Supplementary Table S2 present the perceived effects of gender-based violence on university life among participants who reported such experiences. Outcomes were assessed using ordinal Likert-type items from 1 to 5 points, with higher scores indicating a greater perceived impact. Results are reported as medians and interquartile ranges [IQR], and subgroup comparisons were conducted using Kruskal-Wallis tests according to age group, academic rank, and contract type.

**Table 5:** Effects of gender-based violence on university life.

Outcome	Group	Median [IQR]	*p*-value
Physical discomfort	Young	5 [4–5]	0.800
	Middle adult	3.5 [3–4.75]	
	Older middle adult	5 [3–5]	
	Low rank	5 [4.5–5]	0.394
	Middle rank	3 [3–4.5]	
	High rank	4 [3.5–4.5]	
	Fee-based	5 [4.5–5]	0.195
	Temporary contract	5 [3–5]	
	Permanent contract	1 [1–1]	
Psychological distress	Young	3 [2–4]	0.302
	Middle adult	4 [3–5]	
	Older middle adult	4 [2–5]	
	Low rank	3 [2–4.5]	0.382
	Middle rank	5 [3–5]	
	High rank	3 [3–4]	
	Fee-based	2 [2–3]	0.035
	Temporary contract	4.5 [3.25–5]	
	Permanent contract	3 [2.5–5]	
Isolation from peers/work team	Young	3.5 [2.25–4.75]	0.680
	Middle adult	4 [3–4.75]	
	Older middle adult	4 [3–5]	
	Low rank	4 [2.5–5]	0.605
	Middle rank	4 [3.75–5]	
	High rank	3 [2.75–3.5]	
	Fee-based	4 [4–5]	0.304
	Temporary contract	3 [2.75–5]	
	Permanent contract	4.5 [4–5]	

Higher scores indicate a greater perceived impact. Values are presented as median and interquartile range [IQR]. *P*-values correspond to the Kruskal-Wallis tests comparing age groups, academic rank categories, and contract types.

Table 5 shows the effects of gendered violence on university life. Psychological distress was significantly associated with the contract type (*p* = 0.035). Participants with temporary contracts reported the highest median score, followed by those with permanent contracts, while those with fee-based contracts reported the lowest median score. This finding indicates that contract type is associated with the perceived psychological impact of gender-based violence, where academics with temporary and permanent contracts reported the greatest distress. Another effect is related to behavioral changes, specifically changes in travel routes, which shows a significant relationship with the level of academic ranking (*p* = 0.033), presenting the highest medians in the high and medium academic ranges.

Supplementary Table S2 extends the analysis by describing additional behavioral and professional adjustments following experiences of gender-based violence, including changes in career or academic units, routes or commuting patterns, and phone numbers or social media. Changes in routes or commuting patterns showed high values across groups, with a statistically significant difference between subgroups according to academic rank, with mid- and high-ranking academics reporting maximum scores. These outcomes reflect the avoidance and disruption of ordinary academic routines.

Taken together, Tables 5 and Supplementary Table S2 indicate that the effects of gender-based violence extend beyond the immediate event and affect several dimensions. The most robust statistical evidence was observed for psychological distress by contract type and changes in routes or commuting patterns according to the academic rank. These findings support an organizational interpretation of gender-based violence in academia, in which the consequences are not limited to individual discomfort but also involve adaptive behaviors and modifications of work-related routines. Because the analyses were based on ordinal outcomes and modest subgroup sizes, they should be interpreted as exploratory evidence of perceived impacts rather than causal effects.

#### 3.1.3 Analysis about the request for help and reporting mechanisms in the face of any gender violence situation

This subsection describes the sources of support sought by female academics in response to gender-based violence, disaggregated by contract type, academic rank, faculty/unit, and age.

In all types of contracts, the most frequent option was “Colleague men and female academics or lecturers at the University,” followed by “Nobody.” Support outside the university, such as institutional options such as “Authorities or degree headship” and “University officers” were selected less frequently.

Regarding the filing of formal complaints, only 7% of those who reported experiencing violence formally reported the incidents to the relevant units within their university. The 93% who did not report indicated that the most frequent reasons for not reporting were: “What happened to me was not serious enough to report it” (49%) and “I do not trust the bureaucratic processes of the university” (22.0%) (Figure 3).

**Figure 3:**
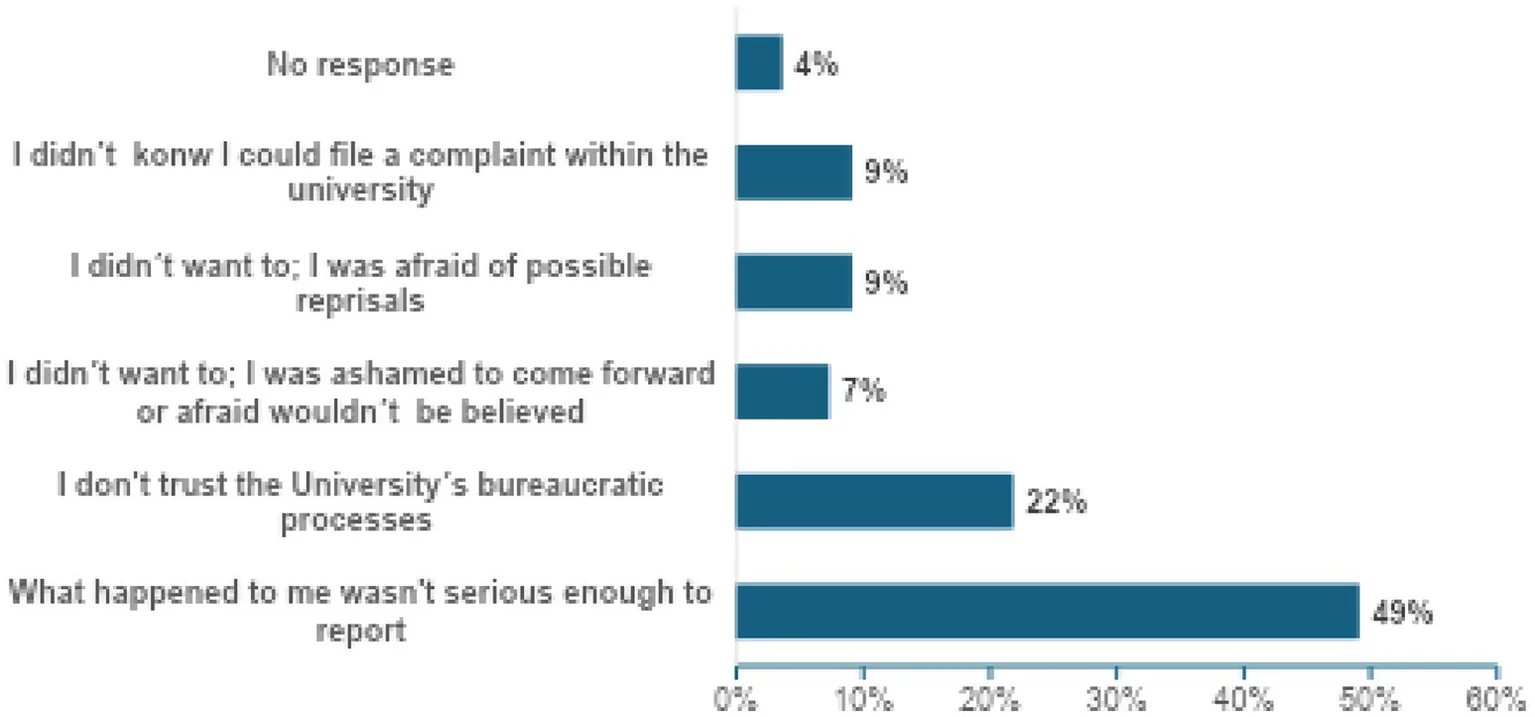
Reasons for the non-reporting of gender-based violence among female academics.

### 3.2 Knowledge about the regulation and perception of institutional response in cases of gender violence

This subsection examines female academics’ knowledge of institutional regulations and their perceptions of institutional responses to gender-based violence. Most respondents reported awareness of the university’s Department of Equality and Gender (93%) and Gender Equality Policy (76%).

Regarding the type of institutional response considered most appropriate in cases of harassment, violence, or gender discrimination, multiple responses were allowed and analyzed as non-mutually exclusive responses. The options with the highest incidence were psycholegal support (80%), awareness activities (77.6%), and physical and mental health support (75.3%).

### 3.3 Institutional culture regarding gender-based violence

Tables 6 and Supplementary Table S3 present the perception of organizational culture concerning gender-based violence. The results were assessed using ordinal Likert-type items from 1 to 7, where higher scores indicated greater agreement with the statement expressed in the item. The results are presented as medians and interquartile ranges (IQRs), and comparisons between subgroups were made using Kruskal-Wallis tests according to age group, academic level, and contract type.

**Table 6:** Institutional support and responsiveness.

Group	Median [IQR]	*p*-value
Young	4.75 [3.50–5.50]	0.122
Middle adult	3.75 [3.19–5.38]	
Older middle adult	5.25 [4.25–6.25]	
Low rank	4.75 [3.44–5.75]	0.492
Middle rank	5.00 [3.50–5.54]	
High rank	5.50 [3.62–6.44]	
Fee-based	5.12 [3.94–6.06]	0.343
Temporary contract	4.75 [3.50–5.25]	
Permanent contract	5.12 [3.69–6.06]	

Median [IQR] values and Kruskal-Wallis *p*-values.

An exploratory reliability analysis was conducted. Four items of the organizational culture were grouped into an Institutional Support and Responsiveness scale, including: institutional awareness that gender-based violence is unacceptable, knowledge of where to seek help within the university, perceived institutional support for affected individuals, and perceived fairness of institutional measures and sanctions. Internal consistency was assessed using Cronbach’s alpha and McDonald’s omega. Reliability was considered adequate (Cronbach’s α = 0.747; McDonald’s ω = 0.768), and a composite score was computed as the mean of the four items, with higher values indicating more favorable perceptions of institutional support and responsiveness to the GBV.

Table 6 presents the distribution of the Institutional Support and Responsiveness Scale according to age, academic rank, and contract type. The participants reported moderate perceptions of institutional support, with median scores ranging from 3.75 among middle-aged academics to 5.50 among those holding higher ranks. However, no statistically significant differences were observed according to age group (*p* = 0.122), academic rank (*p* = 0.492), or contract type (*p* = 0.343).

These findings suggest that perceptions of institutional support and responsiveness are relatively homogeneous among female academic staff. Simultaneously, the observed medians suggest moderate, rather than unequivocally high, confidence in institutional mechanisms.

Supplementary Table S3 presents five individual indicators related to respect for gender and sexual diversity, institutional support, application of fair sanctions, mental health, and community fragmentation. These items were analyzed separately based on age group, academic level, and contract type.

Overall, high median values were observed in all the groups. Statistically significant differences were observed between contract types in terms of respect for gender diversity (*p* = 0.052). The lowest median score of 5.0 was observed for temporary contract staff. Similarly, a significant difference was observed between age and institutional support (*p* = 0.017), with lower median scores of 4.0 found among young and middle-aged academics. A significant difference was also observed between academic hierarchy and the mental health or psychological problems of perpetrators (*p* = 0.024). There is a broad consensus regarding two statements, with a maximum median of 7.0 regarding the fact that gender violence affects and harms the entire university community, and a moderate median regarding the assessment of the fairness of the measures and sanctions adopted in cases of gender violence.

In summary, perceptions of diversity, institutional support, and perpetrators’ mental health varied significantly according to the academic staff profile (age, contract type, or academic rank). This indicates that institutional responses and support are unevenly experienced across different subgroups. On the other hand, there is a broad consensus across all groups on two points: gender-based violence harms the entire university community, and the sanctions applied are perceived as only moderately fair.

### 3.4 Multivariate analysis of factors associated with gender-based violence

Table 7 presents the results of the logistic regression analysis. The variables retained by the AIC-based bidirectional stepwise procedure included the faculty affiliation and contract type. The results from the standard and penalized logistic regression models were highly consistent, indicating that the findings were robust to the estimation procedures.

**Table 7:** Factors associated with reporting at least one experience of gender-based violence: standard and penalized logistic regression models.

Variable	Standard logistic regression OR (95% CI)	*p*-value	Penalized logistic regression OR (95% CI)	*p*-value
Faculty (ref: Social, Business and Law Sciences)				
Science	9.58 (2.23–49.8)	0.004	7.96 (2.00–37.40)	0.003
Humanities	4.24 (1.11–18.4)	0.042	3.77 (1.04–15.23)	0.043
Engineering	12.60 (2.65–79.6)	0.003	9.88 (2.28–54.49)	0.002
Contract type (ref: Fee-based)				
Temporary contract	3.60 (1.04–13.6)	0.048	3.25 (1.00–11.44)	0.051
Permanent contract	1.42 (0.36–5.78)	0.614	1.37 (0.37–5.21)	0.635
N	85		85	
McFadden pseudo-R^2^	0.180		–	
Likelihood ratio test *p*-value	0.003		0.003	

Odds ratios [OR] and 95% confidence intervals [95% CI] are reported for standard and Firth penalized logistic regression models. The reference categories were Social, Business and Law Sciences for faculty and fee-based employment for contract type. Variables were selected using bidirectional stepwise selection based on the Akaike Information Criterion [AIC].

Compared with female academics from the Faculty of Social, Business, and Law Sciences, those from Science (OR = 7.96, 95% CI: 2.00–37.40, *p* = 0.003), Humanities (OR = 3.77, 95% CI: 1.04–15.23, *p* = 0.043), and Engineering (OR = 9.88, 95% CI: 2.28–54.49, *p* = 0.002) exhibited significantly higher odds of reporting at least one experience of gender-based violence. Female academics holding temporary contracts also showed higher odds of reporting gender-based violence than those employed on a fee-based basis (OR = 3.25, 95% CI: 1.00–11.44), although this effect reached only borderline significance (*p* = 0.051). In contrast, age group and academic rank were not retained in the final model, suggesting that disciplinary context and employment conditions (contract type) were more strongly associated with experiences of gender-based violence than individual characteristics or the formal academic hierarchy.

Overall, these findings suggest that experiences of gender-based violence are unevenly distributed across academic contexts and may be shaped more strongly by organizational and employment-related factors than by age or academic rank. However, given the cross-sectional design of this study, these associations should be interpreted descriptively rather than causally.

## 4 Discussion

Against the backdrop of extensive international research documenting gender-based violence in higher education, this study examines its dynamics, perceptions, and institutional underpinnings in the Chilean higher-education context. The most prevalent forms of violence are psychological and sexual violence, with peers being the primary perpetrators. The analysis revealed relevant findings associated with the limited use of formal reporting channels and the predominant incidence of informal peer support networks or the lack of requests for help in situations of gender-based violence. This pattern appears to be driven by institutional mistrust and minimization of incidents, as reflected in the main reasons given for not reporting perceiving the event as “not serious enough” and distrust of the university’s bureaucratic processes. Taken together, these results point to a persistent culture of distrust in which the anticipated personal and professional costs of reporting, particularly under conditions of low hierarchy and contractual insecurity, discourage engagement with institutional mechanisms and may contribute to systematic under-reporting. Institutional responses should prioritize trust-building measures, transparent and accessible procedures, and explicit anti-retaliation safeguards that apply equally across contract types and academic ranks while strengthening safe first-response pathways that facilitate timely referral and follow-up (Nowrouzi-Kia et al., 2024).

### 4.1 Characterization of gender-based violence in the university context and its effects

The findings confirm a high prevalence of self-reported gender-based violence among female academics, with psychological and sexual violence as the dominant typology, exceeding physical violence. Importantly, the analysis identifies statistically significant heterogeneity in recurrent verbal and symbolic microaggressions by academic unit and contract type, indicating that exposure is not evenly distributed across organizational sub-contexts. This pattern aligns with both national and international evidence showing that psychological and symbolic forms of violence are among the most frequent in university environments (Carrasco, 2018; Universidad Austral de Chile, 2018; Universidad de Chile, 2019; Universidad de Los Lagos, 2019; Diéguez Méndez et al., 2020; Universidad de La Frontera, 2020; Universidad de Aysén, 2021). Prior Chilean diagnostics also reported substantial impacts on staff, underscoring that psychological violence is not a “minor” expression of harm but a key mechanism through which gender inequality is reproduced in academic work (Universidad Austral de Chile, 2018; SUR, 2022; Universidad de Santiago de Chile, 2022). The observed convergence between gender-based violence, mental health burden, and professional functioning supports the need for comprehensive prevention and accompaniment policies rather than isolated, complaint-driven interventions.

A central contribution of this study is the identification of a structural vulnerability in the consequences, which is concentrated among academics with paid and temporary contracts. This finding is consistent with evidence documenting the professional impact of gender-based violence in academic settings (Humbert and Strid, 2024a) and supports an organizational perspective in which gender-based violence is sustained by hierarchies and inequality regimes (Jaramillo and Canaval, 2020; Varela Guinot, 2020). In other words, disciplinary environments and employment precarity may operate as institutional “amplifiers,” shaping not only exposure but also in the perception of the viability of the response to situations of gender violence.

The observed associations, especially when the perpetrator belongs to the academic stratum, suggest the role of disciplinary cultures in regulating tolerance thresholds for violent situations and the perceived costs of reporting. Likewise, the specific vulnerabilities of temporary and fee-based staff in everyday teaching interactions demonstrate that institutional protection and support may be perceived as being weaker. These patterns reinforce broader claims that universities can function as unsafe environments for women (Jackson and Gray, 2025) and support calls to shift from predominantly punitive and individualistic approaches to institutional models that prioritize care, accompaniment, and accountability (Cagliero et al., 2025).

Contextual distribution is also important. The occurrence of episodes of violence clusters in the core spaces of academic life classrooms/laboratories, offices, and campus areas, while virtual environments are present but less significant. This concentration is consistent with evidence that psychological violence and harassment are frequent and repetitive in everyday academic interactions (Humbert and Strid, 2024b). Structurally, such patterns are expected in organizations characterized by strong hierarchies, competitive climates, and high job insecurity, all of which can intensify employees’ exposure to workplace bullying and promote silence. In Chile, these dynamics are compounded by neoliberalized academic governance, which prioritizes productivity and increases the workload pressure on female academics (Araneda-Guirriman et al., 2025).

Finally, the predominance of non-reporting and reliance on informal networks, explained mainly by mistrust in bureaucratic processes and event minimization, reveals a critical gap between formal regulations and institutional trust. This finding resonates with the “culture of silence” described in university settings (Östergren et al., 2025) and with the notion of “network silence,” which emphasizes the relational and shared belief systems that sustain inaction (Pilinkaite Sotirovic et al., 2024). This aligns with the framework of Institutional Betrayal (Smith and Freyd, 2014), where organizations harm those who depend on them through omissions or ineffective responses.

### 4.2 Knowledge about the regulation and perception of institutional response in cases of gender violence

The analysis of institutional regulation revealed a high degree of knowledge of the Department of Equality and Gender and lower knowledge of the “Comprehensive Gender Equality Policy,’ in addition to the low level of formal complaints to the relevant units reporting a low willingness to use available services, due to a lack of trust in institutional bureaucratic processes and the potential subsequent consequences for well-being and job performance (Kim et al., 2020).

This finding should be interpreted within the context of the Chilean post-2018 feminist mobilization and the subsequent expansion of institutional mandates to address harassment, violence, and gender discrimination in higher education (Iuspa Santelices, 2021; Chapa Romero et al., 2022; Suárez et al., 2024), as crystallized by Law 21.369 (BCN, 2021). While Chilean higher education institutions have increasingly formalized internal gender policies (Vergara et al., 2024), our results suggest that the existence of formal instruments is not equivalent to an effective institutional policy. The observed gap points to implementation challenges, communication, accessibility, and procedural legibility, which may undermine the long-term viability and practical uptake of these frameworks. This deficiency is consequential because unclear reporting routes, uncertain timelines, and limited feedback can increase the “anticipated costs” of activating formal mechanisms, thereby reinforcing non-reporting and avoidance (Humbert and Strid, 2024a). In line with prior evidence, such institutional bottlenecks compromise the effectiveness of policies intended to prevent and address gender-based violence (Bondestam and Lundqvist, 2020).

In this context, institutional responses should integrate coordinated support pathways, including psycho-legal support, psychosocial care, medical services, and career/professional advice, while ensuring predictable and transparent procedures that minimize retaliation risks and the burden on complainants (Cabras et al., 2022). Because skepticism about institutional responses disproportionately discourages reporting among those with less power, policy design and implementation must incorporate an intersectional lens to prevent the reproduction of inequalities and to target protection toward those facing higher exposure and reporting costs (Colpitts, 2022; Anitha et al., 2024; Humbert et al., 2025).

### 4.3 Perception of gender violence on community

Post-incident dynamics are structured by disciplinary and organizational contexts: higher reports of violence are concentrated in the faculties of science and engineering, pointing to elevated relational costs and weaker conditions for collective repairs. This is particularly salient given that these units include STEM fields, where the literature documents persistent gender inequality and elevated risks of gender-based violence and backlash dynamics (Hills et al., 2024). In parallel, intersectional research shows that psychological violence and harassment disproportionately affect minority groups and can erode unit functioning, reinforcing the importance of analyzing community impacts beyond aggregate averages (Humbert and Strid, 2024a).

Conversely, the formation of protection and care networks appears more frequent among academics with temporary or fee-based contracts, suggesting that contractual insecurity may encourage informal adaptive support strategies in the absence of trusted, formal routes. While such networks may buffer immediate harm, reliance on informal responses risks uneven protection and differential access to justice across employment strata in the long term. This reinforces the need for formal, reliably implemented institutional responses that reduce underreporting and its cumulative costs (Bondestam and Lundqvist, 2020).

From an organizational perspective, these findings support prioritizing structural reforms that address insecurity, rigid hierarchies, and disciplinary cultures as conditions that shape both harm and recovery in the workplace. In this sense, the hierarchical nature of academic organizations should be leveraged for accountable leadership and institutional change, enabling senior female academics and allies to operate as change agents and defenders of equality (O’Connor et al., 2021; Owusu-Kwarteng et al., 2025). Simultaneously, evidence on policy implementation in neoliberal university settings cautions that guidelines alone are insufficient when institutional sexism and masculinized structures remain intact (Gardiner and Finn, 2022). Therefore, the university’s challenge is to operationalize its regulatory commitments through targeted, unit-sensitive interventions and measurable organizational practices that translate the recognition of harm into lived safety and organizational justice (Bondestam and Lundqvist, 2020; Nowrouzi-Kia et al., 2024).

### 4.4 Perception of cultural aspects associated with gender violence

The findings on organizational culture show pronounced disciplinary heterogeneity. The Faculty of Social Sciences reported higher perceived safety for women, whereas the Faculty of Sciences and Engineering reported lower safety appraisals for women. This contrast may be discussed in relation to campus-level ecology and institutional configuration: variations in location, density, and patterns of social interaction across campuses have been associated with differential risks and climates regarding victimization and reporting in higher education settings (Tashkandi et al., 2022). In addition, leadership appears to be a plausible organizational mediator, as gender composition and institutional leadership characteristics can shape reporting climate and institutional responsiveness (Cook et al., 2023).

At the cross-sectional level, the evidence points to a culturally entrenched “distrust” that coexists with and may be reinforced by employment insecurity. Precarious employment (fee-based positions) is not only a risk condition for exposure but also appears to be a cultural constraint on collective functioning. This dynamic is further reflected in the gap between discourse and institutional practice: while respondents recognize gender-based violence as unacceptable, they report limited confidence in the adequacy of the measures and sanctions. This configuration is consistent with evidence showing that institutional context and intersectionality shape both the disclosure costs and organizational consequences of gender-based violence (Humbert et al., 2025).

These cultural mechanisms have direct implications for institutional learning. This reinforces the need to situate institutional responses beyond isolated protocols and acknowledge the interaction between organizational dynamics and broader legal and social environments, as illustrated by comparative experiences such as those in Spain (Soler Gallart and Joanpere Foraster, 2024). In the Chilean context, the effectiveness of Law 21.369 will depend less on formal compliance alone than on the decentralization of prevention and the conversion of shared responsibility into everyday community and curricular practices, supported by transparent implementation, credible protection guarantees, and unit-sensitive strategies that address disciplinary variation and contractual vulnerability.

### 4.5 Practical implications and organization of main ideas

The international regulatory architecture of CEDAW (1979), the Beijing Declaration and Platform for Action (ONU MUJERES, 1995), and SDG 5 of the UN 2030 Agenda (United Nations, 2018), together with Chile’s higher-education legal framework, provide a consolidated normative basis for advancing substantive institutional change in gender equality and the prevention of gender-based violence. In Chile, Law 20.370 (BCN, 2009), Law 21.091 (BCN, 2018), Law 20.129 and its quality assurance criterion on coexistence, gender equality, diversity, and inclusion (BCN, 2006), and, critically, Law 21.369 (BCN, 2021), explicitly position non-discrimination and gender equality as guiding principles for university governance and management. However, the study’s results indicate that regulatory availability does not automatically translate into effective protection: a persistent implementation gap, visible in underreporting, limited procedural knowledge, and uneven unit climates, requires a shift from formal compliance to operational capacity building and organizational accountability to address it.

Based on empirical evidence, four strategic institutional management axes should be prioritized to reduce female academics’ exposure to and the consequences of gender-based violence, particularly in conditions of contractual vulnerability.

#### 4.5.1 Axis 1: Prevention

Prevention should shift from generic, campus-wide messaging to unit-sensitive and risk-informed actions. This includes (a) targeted interventions by faculty/unit to shift local norms that sustain microaggressions; (b) strengthened institutional communication through segmented dissemination strategies that ensure timely and accessible information, especially for groups showing lower policy awareness; (c) preventive focalization in high-risk everyday academic settings (teaching, supervision, laboratories, offices) where incidents concentrate; and (d) psychoeducational initiatives to counteract distrust by explicitly integrating leadership responsibilities and context-specific indicators for monitoring change over time.

#### 4.5.2 Axis 2: Socialization

Socialization must be treated as an implementation function rather than a one-off dissemination exercise. Priorities include: (a) improving practical knowledge of reporting and support routes (where to go, what happens next, timelines, protections); (b) communicating the regulatory framework and the range of institutional measures and sanctions in plain, actionable formats; and (c) increasing the visibility of procedural outcomes while protecting confidentiality to strengthen credibility and reduce the “anticipated costs” of engaging with formal mechanisms. In contexts where “does not know/no response” dominates perceptions of sanctions, transparency becomes a core prevention tool, not an optional add-on.

#### 4.5.3 Axis 3: Protection

Protection requires strengthening the institutional “first reception” capacity and ensuring the equivalence of safeguards across employment strata. Key actions are as follows: (a) professionalized first reception with traceability, standardized risk assessment, and anti-retaliation guarantees; (b) formalization of trained peer-support networks as safe referral pathways (not substitutes for institutional responsibility); (c) explicit assurance of equal access to protection and support regardless of hierarchy or contract; and (d) specific support measures for fee-paid personnel and other precarious groups, given their higher vulnerability and clearer performance impacts, as observed in the study.

#### 4.5.4 Axis 4: Follow-up

Follow-up must be institutionalized to prevent policy fatigue and “paper compliance.” This entails (a) continuous monitoring with transparent indicators disaggregated by unit and contract type to detect differential risks and unequal effectiveness, and (b) participatory review mechanisms that include staff representation and allow iterative refinement of procedures based on user experience and documented bottlenecks. Without feedback loops, incidents remain isolated events rather than opportunities for organizational learning and climate change repair.

### 4.6 Limitations and projections

This study has four main methodological limitations that should be considered when interpreting its findings.

i) Sample design and subgroup representation: Although non-proportional stratified probability sampling was implemented to balance representation across faculties and contract types, academic rank (hierarchy) was not used as an explicit stratification variable. Consequently, the natural distribution of the sample limited the size of the subgroups, particularly for women in higher academic ranks (full and associate professors). This limitation restricts the statistical precision of stratified comparisons at higher hierarchical levels and may mask the specific institutional dynamics or heterogeneities inherent to high-ranking female academic staff. Future research would benefit from incorporating multistage quota sampling for academic rank to ensure the adequate representation of smaller but analytically critical subgroups (Eniego et al., 2025).
ii) Sample size, non-response bias, and generalizability: The sample size (*n* = 85) and cross-sectional nature of the data limit the overall statistical power for subgroup analyses and hinder the estimation of causal models. Consequently, the results should be interpreted as associative patterns consistent with a cross-sectional approach rather than as evidence of directional or causal effects. Furthermore, because this study is a single-institutional case design focused on a regional university, its external validity and generalizability to other geographic or institutional contexts in the Global South are limited in scope. Finally, the overall response rate of 37.1% introduces a potential non-response bias, with academics in more stable contractual positions (Temporary and Permanent) showing a greater willingness to participate than those in precarious employment arrangements (fees).
iii) Data collection procedures and potential response biases: The data collection strategy introduces two specific behavioral and cognitive risks. First, the survey relied on the retrospective recall of the past 5 years to assess experiences of gender-based violence, which may introduce recall bias regarding the frequency, timing or severity of past events. Second, because the instrument was administered through personal contact on a highly sensitive topic, the data may be subject to potential social desirability effects or underreporting driven by fear of institutional exposure or apprehension about evaluation.
iv) Specifications of the instrument and construct: Cross-validation of the gender violence categories and scale properties within the broader context of Chilean higher education would contribute to strengthening the validity of the measurement and improving comparability with international studies.

Future research should prioritize the following areas:

- i) Methodological specifications: Qualitative and mixed-methods approaches are required to explain the mechanisms underlying underreporting, institutional mistrust, the “rule of silence,” and observed disciplinary differences in the organizational climate (Gallardo-Nieto et al., 2021). Further research could benefit from the implementation of an explanatory sequential design (Creswell and Creswell, 2023). In this design, a second qualitative phase—using in-depth interviews or focus groups—would allow for a deeper exploration of the reasons behind the high prevalence of psychological violence and the mechanisms of the “culture of mistrust” identified in the quantitative phase of the study.
- ii) Population extension: Extending the analysis to include students and administrative staff would enable a genuine organizational account of gender-based violence, including cross-stratum dynamics and institutional conditions that shape exposure, response, and recovery.
- iii) Curriculum and cultural reforms: Research should examine gender-based violence through academic programs and training interventions to address epistemological, pedagogical, and ethical dilemmas while strengthening institutional capacity for prevention (McLean, 2023). In parallel, studies should explicitly analyze how patriarchal power structures and academic production cultures shape female academics’ vulnerabilities and constrain their agency (Ur Rehman et al., 2024).

## 5 Conclusion

This study demonstrates a decisive implementation gap between the existence of formal gender-equality structures and their effective use: while awareness of institutional gender offices is high, formal reporting remains exceptionally low (7%), driven mainly by minimization of incidents and distrust in university bureaucratic processes. Help-seeking is channeled primarily through informal peer networks rather than authorities, indicating insufficient institutional trust to activate organizational learning and corrective actions.

Taken together, these findings support moving beyond individual punitive logics toward an organizational justice approach that treats prevention, protection, and restoration as operational capacities rather than aspirational policies. This requires enforceable anti-retaliation guarantees, transparent and traceable procedures, and equivalent access to protection regardless of contract type, especially for fee-based and other precarious staff, for whom reporting costs are higher and professional consequences are more pronounced. Finally, achieving the aims of Law 21.369 will depend on translating regulatory commitments into everyday community and curricular practices that are sensitive to disciplinary variation and contractual vulnerability, so that policy becomes lived safety and organizational justice for female academics.

## Data Availability

The original contributions presented in the study are included in the article/Supplementary material, further inquiries can be directed to the corresponding author.
