# Supplementary material for: Gender-based violence against female academics in Chile: organizational vulnerability, underreporting, and work-related impact
Source: Front Psychol. 2026 Oct 7;17:1798923. doi: 10.3389/fpsyg.2026.1798923 (PMC13683110; doi:10.3389/fpsyg.2026.1798923)
Supplement: Supplementary file 1 [file Table_1.DOCX]

**Table S1. Structure and response formats of the Diagnostic Survey on Gender-Based Violence**

| **Section** | **Questionnaire item(s)** | **Domain assessed** | **Response format** | **Coding/analytical treatment** |
| --- | --- | --- | --- | --- |
| **1. Sociodemographic and employment characteristics** | 1–5 | Gender identity, age group, university unit/faculty, academic rank, and employment contract | Categorical single-response items | Coded as nominal or ordinal categorical variables. Used for sample description and subgroup analyses |
| **2. Characterization of gender-based violence** | 6 | Experience of gender-based violence during the previous five years | Binary screening item | 0 = no; 1 = yes. This was the primary binary prevalence outcome |
|  | 6.1–6.9 | Type or manifestation of gender-based violence | Multiple-response categorical items | Categories were not mutually exclusive; participants could select more than one response. Results were reported as frequencies and percentages of mentions |
|  | 7 | Institutional group of the person involved | Multiple-response categorical item | Student, academic staff, or administrative/non-academic staff, according to the questionnaire. Categories were not mutually exclusive |
|  | 8 | Context or location in which the situation occurred | Multiple-response categorical item | Academic, administrative, social, campus-based, external, or virtual contexts listed in the questionnaire. Multiple selections were allowed |
| **2. Perceived effects and response to gender-based violence** | 9.1–9.9 | Perceived physical, psychological, academic, occupational, and professional effects | Nine-item ordinal matrix | Five-point scale: 1 = slightly affected to 5 = severely affected; additional response: Not applicable. Items were summarized using medians and interquartile ranges |
|  | 10 | First source of help or support contacted | Conditional categorical item | Administered only to participants who reported an experience. Response alternatives followed the questionnaire |
|  | 11 | Whether the situation was formally reported | Conditional binary item | 0 = no; 1 = yes |
|  | 12 | Reason for not reporting the situation | Conditional categorical item | Administered to participants who did not report the situation. Categories followed the questionnaire response options |
| **3. Knowledge of regulations and institutional response** | 13–15 | Knowledge of institutional gender-equity regulations and perception of institutional response | Binary and categorical items | Responses were coded according to the original alternatives, including Yes, No, and Do not know, where applicable |
| **4. Community effects** | 16–18 | Personal, emotional, cognitive, and collective reactions to gender-based violence | Categorical and multiple-response items | Multiple selections were retained when permitted. Categories were analyzed as non-mutually exclusive where applicable |
| **5. Organizational culture and gender-based violence*** | 19.1–19.14 | Perceptions of safety, equality, gender diversity, reporting, institutional support, and sanctions | Fourteen-item ordinal matrix | Seven-point scale: 1 = strongly disagree to 7 = strongly agree. Items were analyzed individually and/or as a scale only after verifying the coding direction and reliability |
| **Administrative procedure** | Not included among the 19 substantive items | Voluntary participation, omission of questions, and withdrawal | Ethical/administrative procedure | Not treated as an analytical questionnaire item or study variable |

*** Note:**The questionnaire comprised 19 main multipart items organized into five sections. Items 6–12 included conditional questions and sub-items that depended on previous responses. Multiple-response questions were not mutually exclusive; therefore, the sum of selected responses could exceed the number of participants. The Not applicable option was retained for descriptive reporting and excluded from specific analyses when it was not conceptually meaningful. Missing responses were not imputed.

# Table S2: Effects of gender-based violence on university life.

| **Outcome** | **Group** | **Median [IQR]** | **p-value** |
| --- | --- | --- | --- |
| Change of career/unit | Young | 3 [2-4] | 0.739 |
|  | Middle adult | --- |  |
|  | Older middle adult | 5 [3-5] |  |
|  | Low rank | 5 [5-5] | 0.182 |
|  | Middle rank | 1 [1-3] |  |
|  | High rank | --- |  |
|  | Fee-based | 5 [5-5] | 0.411 |
|  | Temporary contract | 5 [3-5] |  |
|  | Permanent contract | 1 [1-1] |  |
| Changes in routes or commuting patterns | Young | 4 [2.5-4.5] | 0.500 |
|  | Middle adult | 4 [3.5-4.5] |  |
|  | Older middle adult | 5 [5-5] |  |
|  | Low rank | 3 [2-3] | 0.033 |
|  | Middle rank | 5 [4.75-5] |  |
|  | High rank | 5 [5-5] |  |
|  | Fee-based | 3.5 [3.25-3.75] | 0.519 |
|  | Temporary contract | 4 [2.5-5] |  |
|  | Permanent contract | 5 [5-5] |  |
| Changes in phone number/social media | Young | 3 [2-3.5] | 0.346 |
|  | Middle adult | 4 [4-4] |  |
|  | Older middle adult | --- |  |
|  | Low rank | 4 [4-4] | 0.346 |
|  | Middle rank | 3 [2-3.5] |  |
|  | High rank | --- |  |
|  |  |  |  |
|  | Fee-based | 4 [4-4] | 0.346 |
|  | Temporary contract | 3 [2-3.5] |  |
|  | Permanent contract | --- |  |

**Note.** Values are presented as median and interquartile range [IQR] for ordinal Likert-type outcomes. Higher scores indicate greater perceived change or adjustment. P-values correspond to Kruskal-Wallis tests comparing age groups, academic rank, and contract type. Dashes indicate insufficient available responses to estimate median and IQR

# Table S3. Gender-based violence and organizational culture.

| **Item** | **Group** | **Median [IQR]** | **p-value** |
| --- | --- | --- | --- |
| The university recognizes and respects gender and sexual diversity. | Low | 5 [4-6.25] | 0.936 |
|  | Middle | 6 [4-6] |  |
|  | High | 6 [5-6] |  |
|  | Young | 6 [4-6] | 0.975 |
|  | Middle adult | 6 [3.50-7] |  |
|  | Older middle adult | 5 [5-6] |  |
|  | Fee-based | 6 [5-7] | 0.052 |
|  | Temporary contract | 5 [4-6] |  |
|  | Permanent contract | 6 [4.25-6] |  |
| Gender-based violence affects and fragments the university community | Low | 7 [7-7] | 0.403 |
|  | Middle | 7 [7-7] |  |
|  | High | 7 [7-7] |  |
|  | Young | 7 [7-7] | 0.985 |
|  | Middle adult | 7 [7-7] |  |
|  | Older middle adult | 7 [7-7] |  |
|  | Fee-based | 7 [7-7] | 0.438 |
|  | Temporary contract | 7 [7-7] |  |
|  | Permanent contract | 7 [7-7] |  |
| The university supports people affected by gender-based violence | Low | 5 [3-6] | 0.572 |
|  | Middle | 5 [2.50-6] |  |
|  | High | 6 [4.25-6] |  |
|  | Young | 4 [3-6] | 0.017 |
|  | Middle adult | 4 [2-5.25] |  |
|  | Older middle adult | 6 [4-6.25] |  |
|  | Fee-based | 5 [4-6] | 0.357 |
|  | Temporary contract | 4 [3-6] |  |
|  | Permanent contract | 5 [4-6] |  |
| The university implements fair measures and sanctions | Low | 4 [2-5] | 0.714 |
|  | Middle | 4 [1-5] |  |
|  | High | 4 [3-6] |  |
|  | Young | 4 [2-5] | 0.693 |
|  | Middle adult | 4 [1-5] |  |
|  | Older middle adult | 4 [1.50-5.50] |  |
|  | Fee-based | 4 [4-6] | 0.225 |
|  | Temporary contract | 4 [1.50-5] |  |
|  | Permanent contract | 4 [1-5] |  |
| Perpetrators act because of psychological or mental health problems | Low | 4 [2-7] | 0.024 |
|  | Middle | 7 [4-7] |  |
|  | High | 2 [1.25-5.50] |  |
|  | Young | 6 [4-7] | 0.867 |
|  | Middle adult | 4 [2.75-7] |  |
|  | Older middle adult | 5.50 [2-7] |  |
|  | Fee-based | 4 [2-7] | 0.259 |
|  | Temporary contract | 6 [4-7] |  |
|  | Permanent contract | 4 [2.50-7] |  |

**Note.** Values are presented as median and interquartile range [IQR]. P-values correspond to Kruskal-Wallis tests comparing age groups, academic rank categories, and contract types. Items are presented in the original questionnaire metric. For this table, higher values indicate stronger agreement with positively worded statements.
